# Supplementary material for: Effect of Selenium and Lycopene on Radiation Sensitivity in Prostate Cancer Patients Relative to Controls
Source: Cancers (Basel). 2023 Feb 3;15(3):979. doi: 10.3390/cancers15030979 (PMC9913686; doi:10.3390/cancers15030979)
Supplement: Supplementary file 1 [file cancers-15-00979-s001.zip › cancers-2133247-supplementary.pdf]

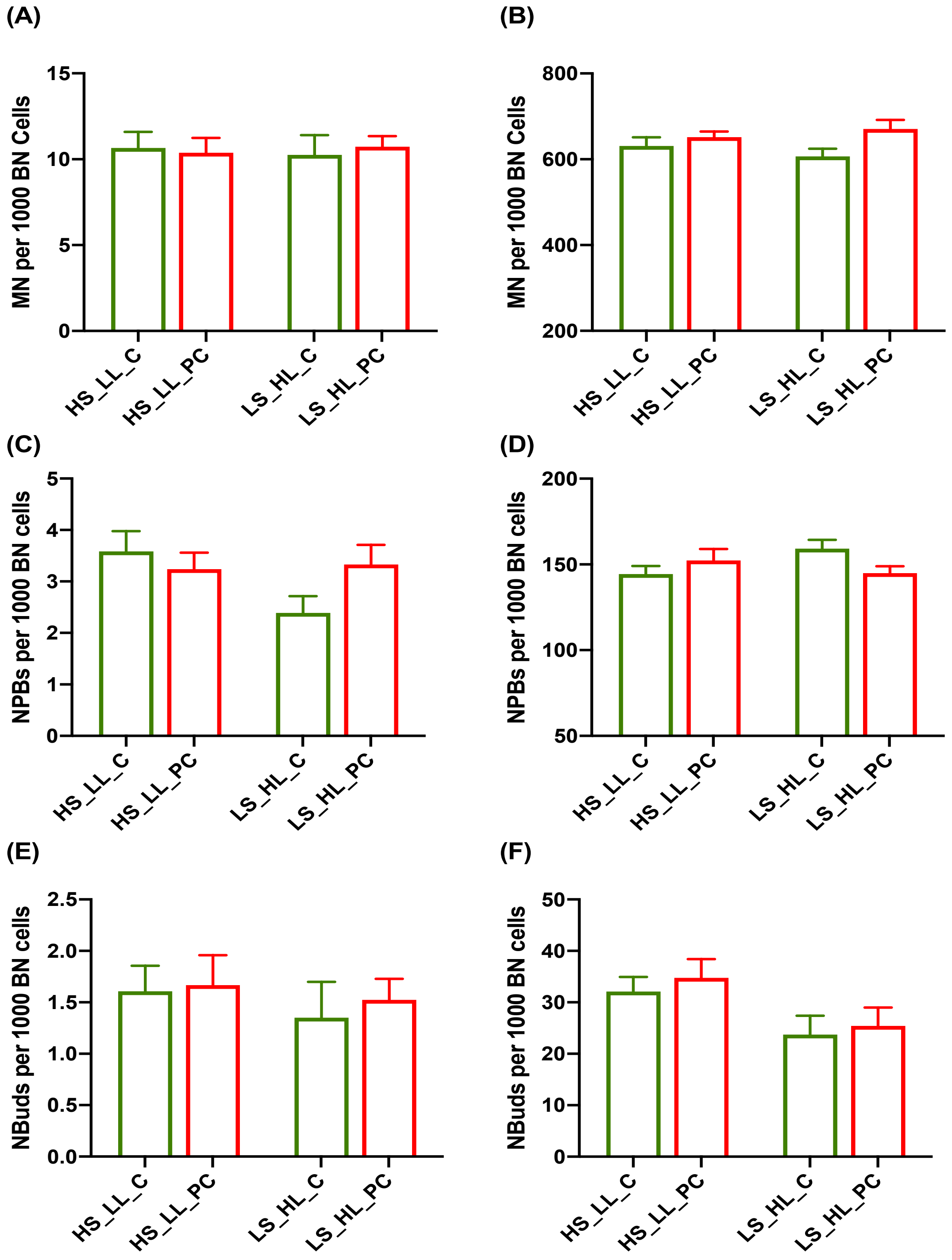

Supplementary Figure S1. CBMN Cytome assay biomarkers in PC patients (red bars) and age matched healthy control (green bars) stratified as per selenium and lycopene concentration (LS: low selenium; HS: High selenium; LL: low lycopene; HL: High lycopene); MN frequency at baseline (A) and after 3 Gy irradiation (B); NPBs at base line (C) and after 3 Gy irradiation (D); NBuds at baseline (E) and after 3 Gy irradiation (F); N= 24, 21, 27 and 20 respectively from left to right for each bar.
